# Obstacles, Opportunities, and Ethical Considerations for Genomic Investigations of Individuals Continuously Hospitalized with Treatment-resistant Schizophrenia

**DOI:** 10.1093/schizbullopen/sgaf019

**Published:** 2025-09-04

**Authors:** Richard C Josiassen, Rose Mary Xavier, Tyler E Dietterich, Matthew K Harner, Dawn M Filmyer, Cassie Houpt, Maya L Lichtenstein, Martilias Farrell, Rita A Shaughnessy, Gabriel Lazaro-Munoz, Jonathan S Berg, Patrick F Sullivan

**Affiliations:** Translational Neuroscience, Philadelphia, PA 19119, United States; Department of Research, UTHealth Houston Cizik School of Nursing, Houston, TX 77030, United States; Translational Neuroscience, Philadelphia, PA 19119, United States; Department of Psychiatry and Biobehavioral Sciences, Semel Institute for Neuroscience and Human Behavior, University of California, Los Angeles, Los Angeles, CA 90024, United States; Translational Neuroscience, Philadelphia, PA 19119, United States; Center for Advanced Biotechnology and Medicine, Rutgers University, Piscataway, NJ 08854, United States; Department of Psychiatry, Rutgers University, New Brunswick, NJ 08901, United States; Translational Neuroscience, Philadelphia, PA 19119, United States; Department of Medical Ethics and Health Policy, Perelman School of Medicine, University of Pennsylvania, Philadelphia, PA 19104, United States; Department of Neurology, Geisinger Health System, Wilkes Barre, PA 18765, United States; Department of Natural and Applied Science, Cheyney University, Philadelphia, PA 19319, United States; Translational Neuroscience, Philadelphia, PA 19119, United States; Center for Bioethics, Harvard Medical School, Boston, MA 02115, United States; Departments of Psychiatry and Neurosurgery, Massachusetts General Hospital, Boston, MA 02114, United States; Department of Genetics, University of North Carolina at Chapel Hill, Chapel Hill, NC 27599, United States; Department of Genetics, University of North Carolina at Chapel Hill, Chapel Hill, NC 27599, United States; Department of Psychiatry, University of North Carolina at Chapel Hill, Chapel Hill, NC 27599, United States; Department of Medical Epidemiology and Biostatistics, Karolinska Institutet, Stockholm, Sweden

**Keywords:** schizophrenia research ethics, treatment-resistant psychosis, copy number variants, *continuously hospitalized*-TRS, rare variants, schizophrenia, decision-making capacity, extreme phenotype sampling

## Abstract

**Background and Hypothesis:**

The overarching objective when studying schizophrenia is the development of generalizable knowledge that improves patient health and/or increases our comprehension of their illness. To fully achieve this objective, investigations need to reflect the *full range of individual variation* found within this heterogeneous population. But individuals committed to state psychiatric institutions have been routinely excluded from research because of concerns that they may not be able to understand or provide adequately informed consent. While reasonable, we believe this approach has enabled policies that support implicit bias, contribute to health care disparities, and limit our knowledge of disease mechanisms and treatment.

**Study Design:**

This article provides brief reviews of (1) ethical considerations when recruiting individuals with severely treatment-resistant psychotic symptoms for research, (2) the impact their condition has on decision-making capacity, and finally (3) we provide a first-hand narrative of our experience conducting a genomic study of involuntarily hospitalized individuals and the opportunities/obstacles we encountered.

**Study Results:**

Evidence from published literature shows that cognitive impairment, rather than severity of psychopathology, is the greatest threat to decisional capacity. Ethical safeguards and practical considerations have been developed, including (1) institutional/local research committee review and approval, (2) confidentiality, (3) informed consent, (4) assessment of capacity, and (5) community engagement. Our experience demonstrates that carefully selected involuntarily committed individuals can be included in research.

**Conclusions:**

With ethical safeguards, these individuals deserve the opportunity to volunteer for research regarding the mental illness that has profoundly shaped their lives—to do otherwise is discriminatory.

## Background of the Study

Historically, individuals with chronic and severe treatment-resistant psychotic symptoms (TRS; commonly defined as “treatment-resistant schizophrenia”) have often been excluded from psychiatric research because of concerns that they may not be able to provide truly informed consent.^(^^1-6^^)^ Their exclusion is especially common when involuntarily committed to long-term psychiatric institutions for their care and safety.^(^^7-10^^)^ The term *severe, extremely treatment-resistant schizophrenia* (SeTRS) was recently introduced by other authors,^(^^11^^)^ however, we prefer to characterize our patients as “individuals with TRS who require long-term hospitalizations” (eg, *Continuously hospitalized*-TRS, CH-TRS). It more accurately reflects their chronic long-term institutionalization. Their exclusion largely stems from interpretations of research principles set forth by The Nuremberg Code,^(^^12^^)^ namely (1) the *protection* of research participants from harm, (2) the importance of *voluntary informed consent*, and (3) the *freedom to withdraw* from participation, and other similar documents.^(^^13-15^^)^ These documents were written in response to past unethical events and hopefully to avoid further ethical lapses.^(^^16^^)^ There are certainly some types of research where no consent is required at all, due to the very low risk, and others where potential risks are high and require very stringent consent. Ethical requirements aim to minimize the possibility of exploitation by ensuring that research subjects are not merely used but are treated with respect while they contribute to the social good.^(^^15^^)^ And individuals with TRS who require long-term hospitalization certainly warrant special protection.

Yet, from a different perspective, the exclusion of people with CH-TRS may be considered unethical. We believe that the importance of conducting research on these individuals is especially great in light of the severity of their personal suffering^(^^17-20^^)^ and the public health consequences of their condition.^(^^17^^,^^21^^,^^22^^)^ Moreover, their exclusion has no doubt hindered development of effective treatments for their condition and contributed to the significant health care disparities seen in this population.^(^^23-25^^)^ These well-intended ethical efforts to protect individuals with CH-TRS may at times lead to their harm.^(^^23^^,^^24^^,^^26^^)^ Along with others,^(^^27-29^^)^ we believe these *real life* consequences create a societal need—an ethical imperative^(^^27^^)^—to perform ethically-grounded scientific studies focused on the etiology, treatment, and prevention for this sizable proportion of the worldwide schizophrenia population.

While the unmet needs of these patients strongly argue for more research, their enrollment into scientific investigations has long been an area of ethical sensitivity and continues to be a source of debate and controversy.^(^^30-32^^)^ Many scientists, funding agencies, Institutional Review Boards (IRBs), and hospital staff (clinicians and administrators alike) simply exclude them from research.^(^^1^^,^^3^^,^^4^^,^^33^^,^^34^^)^ It is believed these individuals, by virtue of belonging to this category of patients, are incapable of providing adequate informed consent and should be peremptorily excluded from scientific participation^(^^35-37^^)^—and it is a persuasive argument. It is obvious that many individuals with CH-TRS experience a reality that can be quite unusual,^(^^36^^)^ and multiple studies have documented that groups of severely and chronically ill psychotic patients do more poorly on measures of decision-making capacity (DMC) than healthy control groups and other patient groups with medical and psychiatric illnesses.^(^^38-42^^)^ With this line of reasoning, vulnerable individuals are successfully protected by simply imposing a “blanket exclusion” from research. Of course, this approach also protects scientists, research sponsors, review committees, and institutions against accusations of patient exploitation. The appeal is understandable—it is a simple and easily implemented solution to an ethically complex situation. However, there is an equally critical ethical imperative to ensure that every individual’s autonomy for decision-making is recognized and respected, even for those with CH-TRS. Inappropriately limiting their autonomy is as much an ethical breach as failing to obtain adequate informed consent. The threshold for impairment that renders an individual incapable of decision-making should reflect a societal judgment about the proper equilibrium between autonomy and protection and must be carefully considered on an individual basis.^(^^32^^,^^43^^,^^44^^)^

The stereotyped consideration of people with chronic and severe psychotic symptoms as having an intrinsic incapacity of decision-making has been seriously challenged,^(^^45-47^^)^ with criticism based upon ethically principled arguments (justice and fairness in the burdens and benefits of research; autonomy of individual participants), as well as empirical evidence related to their decision-making abilities.^(^^1^^,^^2^^,^^5^^,^^9^^,^^35^^,^^42^^,^^48-50^^)^ Here, the seminal work of Carpenter et al.^(^^36^^)^ warrants special consideration. These authors were the first to examine the relationship between psychotic symptom severity and decisional performance in a sample composed predominantly of chronic and severely ill psychotic inpatients using the well-validated MacArthur Competence Assessment Tool-Clinical Research (MacCAT-CR).^(^^51^^)^ As expected, the overall results demonstrated poor group performance. But importantly, the Carpenter findings clearly demonstrate that (1) some individuals with chronic and severe psychotic illness do have adequate decisional capacity for informed consent, (2) decisional capacity can be remediated, and (3) cognition appears more relevant than severity of psychosis in predicting the level of decisional impairment. The evidence from this study and others,^(^^8^^,^^44^^,^^52-54^^)^ including 3 recent high-quality meta-analyses^(^^5^^,^^35^^,^^42^^)^ clearly demonstrate that *cognitive* impairment, rather than severity of *psychopathology*, represents the greatest threat to informed decision-making. Moreover, studies of frequent neuropsychological impairments^(^^29^^,^^36^^,^^41^^)^—particularly in learning, attention, working memory, abstract reasoning, and executive functioning – have demonstrated a relationship between global neurocognitive functioning and impaired decisional capacity, although clear relationships between cognitive domains and DMC have yet to emerge.^(^^44^^)^ Most importantly, the findings *point to the need for individualized assessment*. Carpenter et al. summarized the situation this way:

*“…the proposition that a psychotic person ipso facto should lose decision-making power for research decisions is flawed and stigmatizing. Rather than restrict research participation for categories of patients, emphasis should be placed on assuring that procedures for informing and documenting adequacy of consent are routinely practiced”*  ^(p.538)^.

Consequently, we and others^(^^2^^,^^3^^,^^11^^,^^28^^,^^29^^,^^35^^,^^55^^,^^56^^)^ believe or have shown that a number of people with chronic and severe psychotic symptoms, even those involuntarily committed to long-term institutions,^(^^2^^,^^16^^,^^33^^,^^57^^,^^58^^)^ can have demonstrably adequate DMC for participating in research. Therefore, their “blanket exclusion” is inappropriate. Instead, in the process of study recruitment, DMC should be individually assessed to actually determine *whether or not* a specific individual has sufficient functional capacity to participate—and some will. In alignment with the United Nation’s International Convention of 2006 on the Rights of Persons with Disabilities^(^^59^^)^ and the more recent Clinical Genome Action Plan of 2025 for Justice, Equity, Diversity, and Inclusion,^(^^60^^)^ we are convinced that ethical research can and must be done with these individuals in ways that yield valid results, encourage individual autonomy, and build trust among patients, researchers, and those who care for them.

The demonstration that some individuals with CH-TRS have sufficient decisional capacity is immensely informative.^(^^36^^,^^61^^)^ But, moving to the next step of differentiating between those who have sufficient decisional capacity from those who do not can be surprisingly difficult.^(^^42^^,^^57^^,^^62^^)^ How does an investigator know that a potential subject has the capacity to *use* or *weigh* the information being provided for making an informed decision? When does *decisional impairment* become *decisional incapacity*? What are the criteria? Numerous assessment tools have been developed in an attempt to quantify decisional capacity; Dunn et al.^(^^63^^)^ identified 23 such assessment instruments. Some have been more widely adopted than others, such as the MacCAT-CR^(^^51^^)^ and the 10-item Brief Assessment of Capacity to Consent.^(^^52^^)^ Nevertheless, the issue remains regarding how to assess the validity of consent given by individuals with CH-TRS, that is, the identification of valid tools for accurate assessment of their decisional capacity.^(^^64^^)^ Given that serious questions exist about whether any instrument can adequately gauge decisional capacity without considering contextual and individual factors, the validity of these assessment tools will likely remain imperfect. ^(^^42^^,^^44^^,^^63^^,^^65^^)^ Thus, we have, as Michels observed, a “hodge-podge of practices.”^(^^66^^)^ Clearly, DMC is complicated, and more studies are needed to more fully investigate quantitative and qualitative methods of assessment. Unfortunately, most current publications reporting on this population simply assert adequate decisional capacity, without any evidence for how this was determined—Stroup et al. is a notable exception.^(^^67^^)^

The intention here in this report is to share our 4-year experience of conducting a study entitled *“The Genomics of Treatment-Resistant Psychotic Symptoms”* in collaboration with the state psychiatric hospitals of the Commonwealth of Pennsylvania, the PA State Hospital (PASH) study. It began as a small, unfunded pilot study at Norristown State Hospital, where one of the senior authors (R.C.J.) and his long-standing research team have conducted numerous research projects with this population (such as^(^^68-75^^)^). When external funding was obtained for the PASH study, the scope expanded to include the other PA state psychiatric hospitals. Findings from our PASH study have been recently published,^(^^76^^)^ along with several case studies.^(^^77-81^^)^  Table 1 displays pertinent demographics of the PASH group, which included individuals involuntarily institutionalized for both chronic, severe TRS as well as behavior judged to be of danger to self or others. Importantly, the PASH study demonstrated the *feasibility* of studying individuals with CH-TRS and provided some of the first evidence of a subtle yet potentially relevant link between rare genomic variants and persistent nonresponse to antipsychotic drugs.^(^^82^^)^ Our goal in providing this review is to offer our analysis of various ethical considerations involving this protected population and highlight the scientific opportunities and practical challenges we faced in their recruitment. We illustrate what ethical safeguards might look like in practice and some of the ethical complexities involved in undertaking genomic research with these patients. We hope to inspire others to pursue investigations of these important, yet understudied, individuals.

**Table 1:** Some PA State Hospital (PASH) Demographics (*n* = 509)

**Sex**					
	Males	334		65.6%	
	Females	175		34.4%	
					
**Clozapine exposure**		259		50.9%	
			**Group mean**		**SD**
**Age at recruitment**			54.6 years		21.2
**Duration of illness**			32.4 years		12.6
**Age at illness onset**			21.2 years		8.7
**Duration of hospitalization**			13.4 years		10.1
**Number of hospitalizations**			8.1		8.0

## Over-arching Protection of PASH Participants

When our PASH study began, only minimal work had been done on safeguards specific to genetic research in schizophrenia. 2 contemporaneous papers^(^^11^^,^^56^^)^ were published that focused on genetic variants in individuals with chronic and severe TRS but included only minimal information regarding their assessment of capacity and recruitment methods. Genetic studies in psychiatry are generally considered by review committees to be of *minimal risk*,^(^^83^^)^ although the more recent generation of genome-scale data and providing clinical results introduces many other aspects that have a risk/benefit relationship (such as accuracy of the diagnostic findings, impact of secondary findings, long-term storage of data, deposition of data in public databases for NIH-funded studies, etc.) and should warrant careful consideration. Investigators of genetic anomalies in CH-TRS have much at stake in earning and keeping the trust of research participants and other stakeholders. Their trust, once lost, is not easily regained. The burden of ethical conduct in genetic research substantially depends on the integrity of investigators who must carefully anticipate, appraise, describe, and manage study risks; accurately describe the potential benefits to participants and society; and, importantly, weigh and explain how the study’s risks are justified by the potential benefits.^(^^84^^)^

Our team included several senior specialists in medical genetics, ethics, and schizophrenia who all took an active role in designing the protocol, developing the informed consent form (ICF–see Supplement 1), and conducting the study. Importantly, none of the PASH team provided any clinical services at the cooperating hospitals. In 2019, Roberts et al.^(^^85^^)^ published an important outline of safeguards for ethical genetic research with protected populations (Table 2) many of which had already been implemented in our study (**highlighted in bold**).

**Table 2:** Five Composite Measures of Safeguard Procedures

**Composite 1**
Research review & oversight committees
Institutional Review Board’s review and approval
Institutional Review Board’s reviewing research records
Data Safety and Monitoring Board
Reporting adverse events
Conflict of Interest Committee
**Composite 2**
Consent & protection of participants
Informed consent of research participants
Alternative decision maker
**Composite 3**
Confidentiality protections
De-identifying code numbers
Certificate of Confidentiality
**Composite 4**
Community engagement
Meeting with community members
**Composite 5**
Training for researchers^a^

^a^All PA State Hospital (PASH) staff members were trained by the PI at Norristown State Hospital to conduct patient interviews and hospital chart reviews, utilize the Diagnostic and Statistical Manual of Mental Disorders (DSM-IV) to confirm chart diagnoses, administer the Positive and Negative Syndrome Scale for Schizophrenia,^101^ collect vital signs, and administer other study specific assessment tools. The PASH team had worked together for several years and were well trained before PASH was launched. With adequate support we would certainly have augmented our staff to perform additional rating scales (eg, MacArthur Competence Assessment Tool-Clinical Research) to document each individual participant’s level of capacity, assess phenotypic characteristics, and establish a realistic structure for the return of results to participants, their families, and treatment team. 2 NIMH applications for additional support were not reviewed.

Patient protection was in place before any study procedures commenced **(SAFEGUARD #1)**. These initial protections involved review and approval from: (1) 2 federally-registered IRBs, (2) the Medical Director/Chief Psychiatric Officer at the Office of Mental Health and Substance Abuse Services (OMHSAS) of the Commonwealth of Pennsylvania, (3) a local research committee at each hospital site, and (4) “buy-in” from treatment teams on each nursing unit where the study was to be conducted. With 2 IRB approvals in place, the decision to approve research within the state-wide PASH system moved to the Medical Director/Chief Psychiatric Officer of OMHSAS with input from other officials, all of whom are appointed by and represent the Governor of the Commonwealth of Pennsylvania. Their singular responsibility is cultivating a climate of beneficence that results in safety and high-quality clinical care for this severely ill population. They are sensitive to anything that might disrupt patient treatment, introduce more than minimal risk, or be perceived as patient abuse. Regular communication with these officials is essential **(SAFEGUARD #1).**

The next layer of oversight involved approval by a local research committee at each hospital site. These committees were generally composed of the Chief Medical Officer, Chief of Psychiatry, Director of Medical Records, unit nurses, a patient (consumer), a Consumer Advocate, and occasionally a hospital chaplain and/or members of the local National Alliance on Mental Illness (NAMI) **(SAFEGUARDS #1 & #4)**. While members of IRBs and institutional committees base their approval predominantly on interpretations of federal regulatory policy and other ethical codes, these regulations often provide little or ambiguous practical guidance for involving individuals with CH-TRS in research.^(^^32^^,^^84^^)^ Here, the role of the local research committees was critical. Their concerns went beyond ethical issues to considerations of how the PASH team related to health care workers, patients, and, in some cases, even families. These are critical practical considerations that were decisive in terms of whether the PASH study would be successfully implemented. They were authorized to grant site-specific approval based on practical considerations, *real*-world knowledge of long-term institutionalization, and direct knowledge of their hospital inpatients, evaluation of the research team, and their understanding of the PASH study. Meetings with these local committees began with a review of the study protocol and ICF, but the discussions often turned to expressions of concern that the already overworked clinical staff would be asked to “do more” work, and issues of patient protection (See TEXT BOX #1). Some local members implied that our research team might display a degree of intellectual arrogance or entitlement that was dismissive of the clinical staff. Racial concerns were voiced. We could easily have assumed that these issues were insurmountable. However, we believed that we could demonstrate our “trustworthiness” with local committee members, clinical staff, and the patients themselves. These local committees had full authority to approve or decline study participation at their facility. 5 of the 6 PASH institutions did approve our study. Unfortunately, the local research committee at the sixth hospital did not communicate following our meeting.

Box 1 Common questions/concerns raised at local research committee meetings.

• Concerns over disruptions of therapeutic relationships or intrusion into daily clinical activities.

• Questions regarding clinical implications of genomic results and who was responsible to deal with them—no local hospital staff members were trained in genetic medicine and the PASH system did not have genetic counselors.

• Vague concerns about the Health Insurance Portability and Accountability Act I(eg, “What about HIPAA?”)

• Could DNA results cause participants to lose their insurance or be transferred to a different hospital or discharged.

• Might DNA findings lead to malpractice.

• Could DNA findings result in questions of paternity.

• Would the “return of genetic results” be traumatic.

• The story of Henriette Lacks and her family was raised several times as an example of good scientific intentions gone wrong, as well as references to the Tuskegee syphilis and Willowbrook hepatitis studies and the history of eugenics—general mistrust of genetic research.

• Concerns over disruptions of therapeutic relationships or intrusion into daily clinical activities.

Next, with local hospital approval, we were introduced to the staff of each hospital unit. Once introduced, we were “on our own” to cultivate a collaborative relationship with each treatment team **(SAFEGUARDS #1 and #4)**. These teams had no formal approval authority, but their engagement was essential for the success of the study. They were diverse in age, educational backgrounds and levels of interest in psychiatric research. They were deeply invested in caring for and protecting “their” patients and their *protective instincts* were palpable. At each introductory unit meeting, we provided a written summary of the study protocol, an ICF, and a scientific paper pertinent to our discussion.^(^^86^^)^ We discussed the (1) purpose of study; (2) our commitment to the primacy of clinical activity on the unit over our research activity; (3) recruitment approach; (4) a request for input from the clinical team regarding each potential participant; (5) space allocation; (6) access to patient charts; and (7) an explicit request that treatment team members bring any study-related concerns directly to the research team, and, if necessary, to the local research committee **(SAFEGUARD #1)**. When actively recruiting, we requested the opportunity to briefly attend each “morning report” to review recruitment progress, answer questions, and discuss specific potential participants. We remained only for this part of “morning report”. We believed “trustworthiness” could be built in these meetings if we showed competence, honesty, transparency, consistency, compassion, the authentic respect we held for the mission of the state hospital, and our gratitude for being allowed to conduct our investigation in their space.

Our efforts in relationship building reflected both ethical and practical considerations and were mostly met with a positive response, although a few units avoided working with us (eg, “our unit is in transition right now” or “we don’t think any of our patients meet your criteria”). Although our project had received all the necessary formal approvals, these treatment teams retain tacit authority over all activity on their units, and their cooperation was essential for our success.

## Voluntary Informed Consent

What makes human research ethical? Obtaining a signed informed consent is the all-too-common response. A signed informed consent is necessary (in most cases), but in no case is it sufficient for ethical human research. The purpose of informed consent is to ensure that potential participants understand and control whether they enroll in research and participate only when the research is consistent with their values and interests **(53)**.

Ensuring *informed* consent involves a complex interpersonal interaction that requires more than simply reading a document and signing it. Asking questions and active listening are essential. For individuals with CH-TRS, special care must be taken to ensure that their participation is truly *voluntary* and *informed*  **(SAFEGUARD #2)**. This can be especially challenging when potential participants are individuals for whom reality can be experienced quite differently than it is for researchers and clinicians.^(^^30^^)^ With this population, more questions are often needed to clarify an individual participant’s thinking and to identify those needing more time or information during consent discussions.

Regarding *voluntariness,* many ethicists have expressed legitimate concerns that members of protected populations can be easily coerced or pressured into study participation. Appelbaum et al.^(^^87^^)^ provided a valuable model for evaluating voluntariness, coercion, and undue pressure. They argue that a decision is involuntary if it is subject to a “particular type of influence that is external, intentional, illegitimate, and causally linked to the choice of the research subject.” Individuals whose decisions are regularly subject to external authority—such as a prison inmate—may agree to participate due to the illegitimate influence of explicit or imagined threats of punishment for failure to do so or an assumed promise of reward for agreeing. Such consent would not be considered voluntary. Another concern might be that individuals who are *involuntarily* committed for psychiatric care cannot *voluntarily* assent to participate in a research study—that involuntary commitment strips them of their capacity for voluntary decision making. We believe that this opinion is mistaken.

While situational circumstances—such as poverty, life-threatening medical illness, prison or, as in the case of our study, involuntary institutionalization—can influence or limit the range of an individual’s choices, such constraints do not make all their choices involuntary. For instance, certain items of clothing are not permitted on a hospital unit due to the risk of self-harm (ie, belts, sashes), but involuntarily committed individuals still make daily decisions about what clothing to wear. Hospital units may be locked, but individuals can still choose to be involved in daily activities away from the unit, such as art or music therapy, exercise groups, or even semi-skilled employment at a sheltered workshop. Many retain the right to vote in state and national elections. PASH sites grant permission for approved individuals to walk unescorted around hospital property for specified periods of time. Additionally, patients meet monthly with their treatment teams to discuss specific treatment plans, which can nurture a sense of personal agency regarding their own health care. For the PASH study, potential participants were not instructed to volunteer, nor were they threatened with punishment or enticed by offers of financial reward or special treatment during recruitment **(GUIDELINE #2)**. Instead, a simple invitation was extended at a unit meeting, and individuals *self-selected* for study participation.

To make an *informed* decision, participants must be able to understand and weigh the *risks and benefits* of a research protocol. The risks of our PASH study were considered *minimal* by both IRB committees, as they were primarily limited to the discomfort of a single blood draw. Roberts et al.^(^^88^^)^ found that people with schizophrenia do not assign greater risk to venipuncture associated with genetic testing compared to regular blood work. But there were concerns expressed by a few potential participants, such as: (1) would their DNA be cloned into an identical twin; (2) would FBI, CIA, or other agencies have access to their DNA results; and (3) could their DNA results cause them to be transferred or discharged from the hospital. Some staff and potential participants expressed concerns that genetic testing (even in the context of research) may have a negative effect (eg, increased anxiety or depression), or imply to the patient that their clinical condition is immutable.^(^^89-91^^)^ Importantly, every concern was directly addressed, and in very few cases (<10 cases) did the concerns result in non-participation.

While understanding the *risks* of our PASH study was minimal and relatively straightforward, understanding the potential *benefits* of our genomic study was more complex. Genetic testing has evolved rapidly in recent years and provided important new information about diagnosis, treatment, and prevention of certain specific diseases. Despite widespread optimism that genetic research may soon elucidate mechanisms of psychiatric disorders and lead to newer and better therapies, there is still insufficient evidence to support genetic testing as part of standard clinical practice in schizophrenia.^(^^92^^,^^93^^)^ This optimism, when expressed by potential participants, can lead to an inaccurate expectation that research participation will automatically lead to personal benefit or improved personal care—the *therapeutic misconception*.^(^^94^^)^ Patients who have been institutionalized for extended periods may develop the perception that all hospital activities are focused on advancing and protecting their personal health and well-being. While the future of psychiatric genetics appears promising, much of the current knowledge remains academic and highly technical, and not yet translatable into practical and ethically sound guidance.^(^^86^^,^^93^^)^

What then constituted being informed about the possible *benefits* of participation in our genetic study? How much of this evolving body of technical information can and/or should be provided to a potential participant, and to what degree should that information be understood and retained? Little empirical evidence exists regarding any threshold of genetic knowledge that qualifies as ethically sufficient for informed study participation (or even for review committee decision making). Our approach was simple and straightforward. We began with an elementary presentation about cells in the human body, where DNA exists, how DNA functions as a “blueprint” for the ways we develop as unique human beings, and how things can go wrong. From this basic information, we emphasized *2 key points*: (1) the PASH study was an exploration regarding the prevalence of highly penetrant genetic variation—be that in the form of copy number variants (CNVs), or in the case of 1 participant, a trinucleotide repeat in HTT,^(^^77^^)^ and (2) the likelihood of uncovering clinically useful (actionable) information was low. We explained that our primary aim was academic, although we hoped that our findings would one day become the basis for future development of novel treatments. During recruitment, we welcomed any clinical, technical, and/or hypothetical questions and attempted to address each question with understandable information. We wanted our interactions to be educational, but we believed our study did not require sophisticated genetic knowledge for participants to make an informed decision. What mattered was that the participants had “adequate information” that aligned with the level of risk associated with the research protocol. We emphasized that our study was “exploratory” and constantly worked to diminish unrealistic expectations that clinically meaningful (actionable) information would be uncovered.

## Determination of Individual Decisional Capacity

While informed consent is at the heart of ethical human research,^(^^15^^)^  *meaningful* informed consent is possible only when the person giving their consent has the capacity to *use or weigh* the information being provided in making their decision. To begin, it is important to distinguish between the concepts of *capacity* and *competency*. Capacity describes a person’s ability to make a specific decision regarding treatment or research participation, whereas competency is a global assessment and a legal determination made by a judge in court.

Box 2 Assessing decisional capacity.^42^

***Dimensional scores**:* use of structured tools to psychometrically assess performance within individual domains of abilities deemed core to DMC (such as the “four factor model” of the *understanding*, *appreciation, reasoning, and expressing a choice*) to return a score for each dimension, such as the highly influential MacArthur Competence Assessment Tool-Clinical Research (MacCAT-CR).

***Cut-off standard**:* applying a cut-off score or scoring algorithm usually derived from a rating scale, such as the 10-item University of California, San Diego Brief Assessment of Capacity to Consent–UBACC).

***Expert clinical judgment**:* expert opinion returning a binary judgment (*yes* or *no*) that *may* or *may not* be guided by ancillary information from evaluative tools.

Assessing capacity to consent for research has been measured in 1 of 3 ways^(^^42^^)^ as shown in TEXT BOX #2. Each approach has advantages and limitations. Despite its complexity, a cursory look at the relevant literature finds that most evaluations of DMC capacity are still based on expert clinical judgment, without mention of standardized tools or discussion of criteria—even though this approach is vulnerable to well-documented biases.^(^^95^^,^^96^^)^ One study^(^^65^^)^ found only a moderate level of interrater reliability for binary capacity judgments made by expert clinicians (mean kappa of 0.60), although when transcripts of structured interviews were provided, agreement among expert clinicians markedly improved (mean kappa of 0.84). A very recent and authoritative meta-analysis^(^^5^^)^ stated that a “good DMC evaluation” should: (1) include both dimensional methods and expert judgment, not structured assessments alone; (2) be brief, as more concise information may help patients to demonstrate capacity; and (3) be done when the patient’s symptomatology has stabilized. The recent report from Spencer et al.^(^^2^^)^ illustrated the complexity. These authors assessed DMC for research in severely ill psychotic inpatients and found that ~50% of their sample demonstrated adequate capacity when using a categorical outcome (*yes* or *no)* based on the standard of *expert clinical judgment*. Initially, these investigators intended to use the MacCAT-CR as the basis of determining decisional capacity, but ultimately determined that using a quantitative measure alone does not necessarily inform decisional capacity, which is inherently binary.

How then did the PASH team meet this ethical responsibility? To begin, the PASH sample consisted entirely of individuals who self-selected for study participation. On each hospital unit, inpatients and clinical staff were invited to a special morning meeting to introduce the PASH study – the novelty attracted most of the inpatients. The PASH team was introduced by the head nurse, who importantly emphasized that our team was conducting a scientific study and not providing any clinical care. The Principal Investigator (PI) gave a brief and simple presentation about the study, making ample space for questions from patients and staff, and then offered an invitation to participate in the study. Individuals who volunteered then accompanied the PI (or other PASH team members) and a patient advocate (or unit nurse or aide) to a semi-private location where the protocol and ICF could be presented in detail, without interruption. Had the PASH study been suitably funded, a senior research nurse could have played a central role in the recruitment and assessment process. When recruiting minority participants, a unit nurse, aide or patient advocate of the same racial background usually took part in the consenting process. The ICF was read aloud, 1 page at a time, with time allocated for discussion. The PI asked pertinent questions to assess the participant’s understanding of the content of each ICF page. When all the questions for a given page had been answered, the participant was asked to initial the page.

During the ICF presentation, each participant was also asked questions derived from both the Grisso and Appelbaum “four-factor model” of capacity^(^^97^^)^ and the UBACC^(^^52^^)^ to assess their capacity to consent (see Table 3). The assessment included closed-ended *yes/no* questions (eg, “Is anyone (nurse, doctor) making you do this test?” or “Will you get paid for giving your blood?”) and *open-ended* questions (eg, “Why do you think you belong in this study?” or “Can you tell me in your own words if this study will benefit you?”). Each participant was queried on the 4 capacity domains throughout the consenting process and again prior to phlebotomy. The questions were not used to gather “correct” answers that would lead to a pre-determined “cut-off” score, but rather to provide the PI and PASH team insight into whether each potential participant had a general understanding of the study. Did they grasp that their decision was voluntary? Could they name any risks associated with the study? Did they understand that they could refuse to participate without any negative consequences? Inquiries into the motivations for participation required more thoughtful exploration. Published findings support the notion that individuals with schizophrenia see the value of CR^(^^98^^)^ and often give reasons for participating that resemble those given by non-psychiatrically ill research volunteers.^(^^99^^,^^100^^)^ Among the PASH participants, many described an altruistic motivation (eg, “it might help patients in the future”) while others simply expressed their appreciation for being asked to join in the study (eg, “I’ve wanted to be in a study but never was asked”). Some seemed motivated by curiosity (eg, “I remember learning about genes back in high school”) or scientific progress (eg, “might lead to making better medicines”). On occasion, their reasoning was irrational, as the woman who believed her blood was *“green and only turned red when exposed to oxygen,”* which she believed could be proven by participating in the study. Perhaps the most common motivation was the increased attention from PASH team members. If any misconception was identified (eg, “It will help me get out of the hospital” or “I will get paid”), the mistaken idea was corrected or clarified, and the participant was asked if they still wanted to be part of the study.

**Table 3:** Legally Relevant Criteria for Decision-Making Capacity and Approaches to Assessment

**Criterion**	**Patient’s task**	**Questions for assessment**
Communicate choice	Clearly state a preferred choice—participate or not participate	Have you decided whether you want to take part in this study? Can you tell me some of the reasons for your choice? Was it a hard choice for you?
Understand the relevant information	Grasp the fundamental meaning of the information communicated	Can you tell me in your own words what the scientist told you about this project? Do you think you belong in the project?
Appreciate the situation and its consequences way?	Acknowledge their mental illness and likely reasons they are being asked to participate	Should you be in this hospital? Do you think being part of this project will help you in any Is this a study about new medication?
Reasons for taking part in the study	Suggest rational reasons for participating	How did you decide to participate in this project? Do you think you will learn something important that could help you?

After reading the entire ICF and dealing with various questions, the potential participant was invited to sign the ICF, followed by the PI and witness providing their own signatures. The signed ICF was xeroxed, with a copy given to the Director of Medical Records and another placed on the participant’s active hospital chart. The original ICF was retained by the PI and assigned a unique subject identification number **(SAFEGUARD #3).** Each duly-executed ICF was filed and retained as a legal study document, even if the potential participant later withdrew or was excluded. Throughout the consenting process, it was reiterated to the participant that the signed ICF gave the PASH team permission to begin reviewing medical and pharmaceutical records, but *did not guarantee* that the patient would be included in the project. The ultimate decision to *include* or *exclude* the potential participant was based on the degree to which an individual patient: (1) comprehended information presented in the ICF; (2) demonstrated capacity across the “four factor capacity model’ of *understanding*, *appreciation, reasoning, and expressing a choice* observed during recruitment; (3) discussion with PASH team members and clinical staff about each participants’ perceived capacity; and (4) a review of medical and pharmaceutical records These discussions regarding capacity usually resulted in a consensus of opinion, although not always. When opinions diverged regarding DMC, the relevant concerns were discussed more thoroughly. Staff opinions regarding insufficient DMC were not overruled by the PI and usually resulted in exclusion.

The final enrollment decision was usually made and communicated within 24 hours. Each potential study participant was again asked whether they wanted to *continue* or *withdraw* from the study at the time of blood collection. The PI met individually with each patient who was *not selected* to discuss the reason(s) for being *excluded* from the study. In most instances, the individuals were indifferent about not being included, although some took it as a positive sign of their improving psychiatric condition. A few participants (<5 cases) reacted negatively to the information, as if they were being rejected. In 2 instances, individuals who were “rejection sensitive” required the services of the unit psychologist to assuage their concerns. For potential participants with involved family members or legal guardians, the PI obtained verbal permission to contact the patient’s family and discuss the study. In some instances, the family wanted to meet with the PI to discuss the project. A few patients who had been declared *incompetent* in a court of law were enrolled with written consent from their surrogate (parent or sibling) and the participant’s verbal assent **(SAFEGUARD #2)**. Early on, we attempted to obtain consent for individuals deemed *incompetent* and overseen by lawyers and judges (surrogates), but these efforts were *never* successful (always ignored), and we soon discontinued our attempts. The PASH team was highly visible, and occasionally, patients who had not accepted the original invitation later approached the PASH team about being included in the study. Ultimately, the original PASH sample consisted of 692 patients who self-selected and signed the ICF. Of these original cases, 183 (27%) were *not included* in the final PASH sample (*n* = 509 cases). Of the excluded cases, 140 either had <5 years of continuous hospitalization, an ambiguous psychiatric diagnosis, <3 adequate antipsychotic drug trials, and/or were considered decisionally impaired; 12 withdrew their informed consent; 10 refused blood work; 1 refused to allow DNA evaluation; and 1, with family guardian approval, refused to give assent for study. Table 4 summarizes a series of actual cases, selected to illustrate different recruitment situations.

**Table 4:** Examples of Ethical Concerns During Recruitment and Their Resolution

**Case #1**—A 73- year-old single white male - born to a single, teenage mother and placed in orphanage. PA State Hospital (PASH) admission at 17 years of age – single hospitalization for 57 years. Symptoms included paranoia, delusions, auditory hallucinations, aggression/irritability, and self-mutilation (surgically removed a testicle). Often put in isolation or physical restraints. Chronic hyponatremia with seizures. For decades excluded from research, but in 2003 enrolled (with PI) in a successful hyponatremia treatment study and transferred to a less restrictive PASH unit, with monitoring by PI (4 years), and no further isolation or restraints. **Concern:** He might think PASH study has clinical benefits as with other study. **Resolution:** During the consenting process PI repeated that PASH was an academic genetic study with little chance of clinical value, which he acknowledged he understood. **Motivation:** Extreme boredom and enjoyed research team attention.
**Case #2**—A 65-year-old single white female - born to parents who were PASH inpatients at time of conception - placed in foster care. She had psychotic symptoms starting at age 9 years. 5 PASH admissions – most recent continuous hospitalization for 22 years. Symptoms included thought disorder, delusions, selective mutism, paranoia, with frequent verbal or physical assault of males. In 2017 she enrolled (with PI) in a successful tardive dyskinesia study. **Concern:** When invited into PASH study she refused stating, “I don’t have any genes!!” **Resolution:** Delusion never challenged. But with time she approached PI on hospital unit with questions about genes, asked to see pictures of genes, and eventually volunteered. **Motivation:** Curiosity and enjoyed research team attention. Simple expressions of altruism.
**Case #3**—A 70-year-old single black male —1 of 11 children. Auditory hallucination at 16 of age, and by 25 he had made 4 very serious suicide attempts, the last in response to command hallucinations instructing him to jump off a bridge onto a highway. 15 acute hospitalizations, 2 PASH admissions—most recent continuous hospitalization for 34 years. At 66 years of age, in response to command hallucinations, set fire to the state hospital ward—moved to a high-security LTSR (24-hour 1:1 or 2:1 supervision—isolated as sole resident). **Concern:** He retained legal competency for medical decisions, but since in isolation his recruitment could be seen as coercive or racist. **Resolution:** PI met with caseworker (a black male) and psychiatrist to discuss possible recruitment. Met with older brother, the only sibling with whom he had any contact—he agreed to discuss PASH with his brother. Consensus reached about attempting recruitment—he gave informed consent. Importantly, the brother insisted he meet again privately with the patient to confirm voluntary consent before any study procedures. **Motivation:** Unknown but actively engaged in conversations with brother and research team about the study. Brother motivated by potential scientific value.
**Case #4—**A 56-year-old single white male—with onset of auditory hallucinations at 16 years. Admitted for acute care, diagnosed with paranoid schizophrenia and catatonic features—treated with antipsychotic drugs (APDs) and electroconvulsive therapy (ECT) and discharged to his family. Between 19 and 25 years, alternated between acute hospital and home, until he consumed 2 bottles of industrial strength acid-based cleaning agent and then found wandering a hometown street in 12-degree Fahrenheit winter weather, shoeless and without a coat. Involuntarily committed to PASH facility—most recent continuous hospitalization of 12 years. There he attempted to remove his eyes (was blinded)—with other acts of self-mutilation. Percutaneous endoscopic gastrostomy (PEG) tube tube due to severe esophageal damage and refusal to eat. Confined to single occupancy room, often 2-point restraints and 24-hour 1:1 and 2:1 observation. **Concern:** Legally incompetent with parental guardianship. **Resolution:** Parents were retired university professors—requested protocol, informed consent form (ICF), and our published papers. Following several conversations they signed the ICF—but with the proviso that son gave his assent. Verbal assent obtained and documented after being read the ICF by the PI. **Motivation:** Parents motivated by potential scientific value—son’s motivation unknown.
**Case #5—**A 63-year-old single black male—graduated with Master’ Degree (PI verified). Onset of severe auditory hallucinations and paranoia at 24 years. 5 acute hospitalizations with one involuntarily commitment to PASH facility—current continuous hospitalization of 24 years. **Concern:** Prior positive research experience with PI and readily consented to participate. But at venipuncture, abruptly withdrew his consent with physical threats. **Motivation:** Believed DNA could be examined by law enforcement. Withdrawn from study.

## Conclusion

The overarching objective when studying individuals with schizophrenia is the development of generalizable knowledge that improves their treatment and/or increases our comprehension of the mechanisms underlying their illness. The human beings who participate are the means to secure such knowledge. To achieve this objective, an integrated analysis that reflects the *full range of individual variation* in this highly heterogeneous population, including those with persistent and severe psychotic symptoms, is necessary. Many severely symptomatic individuals are involuntarily committed to public state psychiatric institutions, where they have been *automatically excluded* from psychiatric research. What is critically needed is not more stigmatization or neglect of this segment of the schizophrenia population, but rather an increased effort to include them in meaningful research with the necessary safeguards to provide safety and protect their rights and autonomy. We are convinced that schizophrenia research can and must begin to include individuals with CH*–*TRS. It does not mean that all individuals with CH*–*TRS should be uncritically offered the opportunity to volunteer, but it does mean that individuals with CH*–*TRS as a group should not be peremptorily excluded. With ethical safeguards and individual assessment, these individuals deserve the opportunity to volunteer for research regarding the mental illness that has profoundly shaped their lives—to do otherwise is discriminatory.

## Supplementary Material

DREXEL_Informed_Consent_sgaf019
