# Supplementary material for: Obstacles, Opportunities, and Ethical Considerations for Genomic Investigations of Individuals Continuously Hospitalized with Treatment-resistant Schizophrenia
Source: Schizophr Bull Open. 2025 Sep 4;6(1):sgaf019. doi: 10.1093/schizbullopen/sgaf019 (PMC12496010; doi:10.1093/schizbullopen/sgaf019)
Supplement: DREXEL_Informed_Consent_sgaf019 [file drexel_informed_consent_sgaf019.doc]

**Drexel University**

**Consent to Take Part**

**In a Research Study**

***1. Title of research study:*** The Genomics of Treatment-Resistant Psychotic Symptoms

***2. Researcher:*** Richard C. Josiassen, PhD

***3. Why you are being invited to take part in a research study***

We invite you to take part in a research study evaluating genetics of individuals who have been treated with various psychiatric medications, but who have had very little (if any) benefit even after years of treatment. You are being asked because we feel that you may be an individual who has not benefitted significantly from standard psychiatric medications.

***4. What you should know about a research study***

- Someone will explain this research study to you.
- Whether or not you take part is up to you.
- You can choose not to take part.
- You can agree to take part now and change your mind later.
- If you decide to not be a part of this research no one will hold it against you.
- Feel free to ask all the questions you want before you decide.

***5. Who can you talk to about this research study?***

If you have questions, concerns, or complaints, or think the research has hurt you, talk to Dr. Richard C. Josiassen at (267) 304-1115.

This research has been reviewed and approved by an Institutional Review Board (IRB). An IRB reviews research projects so that steps are taken to protect the rights and welfare of human subjects taking part in research. You may talk to them at (215) 762-3944 or email HRPP@drexel.edu for any of the following:

- Your questions, concerns, or complaints are not being answered by the research team.
- You cannot reach the research team.
- You want to talk to someone besides the research team.
- You have questions about your rights as a research subject.
- You want to get information or provide input about this research.

***6. Why are we doing this research?***

Many people with psychotic illnesses, like schizophrenia, do not improve clinically with current treatments. Many of these individuals with treatment resistant psychotic symptoms live in state psychiatric hospitals, and have been treated for years, or decades, but have shown little or no clinical improvement. We do not know why some people benefit from psychiatric medications while others seem to have “treatment-resistant psychotic symptoms” and do not seem to benefit at all.

Dr. Josiassen and his collaborators are studying this question in a research project called ***The Genomics of Treatment-Resistant Psychotic Symptoms***. We are inviting you to participate because you have a psychotic illness that has not benefitted from treatment. If you choose to participate, we will obtain a small amount of blood from your arm. We will study DNA from your blood cells to find out if there is a genetic reason why you have not responded to treatment. DNA is the chemical that makes up your genes. Genes are the information used by our cells that affect how we look and function. Genes tell the cells in your body to make different proteins that, along with the environment, affect how our bodies work. The study of many genes at the same time is called “genomics”.

We are looking for rare differences (or variants) in genes that may cause some people not to respond to psychiatric medications. A team of internationally recognized medical, genetic and neuroscience experts will carefully evaluate the results from each person to determine if they have an “actionable” neurological, psychiatric or medical genomic disorder.

These disorders are caused by genetic differences. Some of these disorders might be treated with different therapies or medications. Other disorders could benefit from more frequent screening for a treatable medical condition. In this study, we will do our best to try to find any such differences in the people who choose to participate.

If you choose to participate in this study and if we identify any genomic differences that cause an actionable genetic disorder, we will share these results with you and, with your consent, with your treating physician. You can also choose to not share this genomic information with anyone, including your physician.

***7. How long will the research last?***

We expect that you will be in this research study for 30 minutes.

***8. How many people will be studied?***

We expect about 1000 people from the Commonwealth of Pennsylvania State Hospitals will be in this research study.

***9. What happens if I say yes, I want to be in this research?***

If you agree to participate, you must first consent and sign an informed consent document. By signing this form, you are allowing Dr. Josiassen and his team to review your current and past medical and psychiatric records to determine whether you are eligible to take part in the study.

If you are eligible to participate, medical and psychiatric information from your medical records will be entered into a password-protected database in our computer. Your information will be coded with a unique study identification (ID) number that is linked to your personal information in our database. The link between your name and the study ID number is available to only a small number of Dr. Josiassen’s staff and is protected by a password-protected security system. This ID number will be used to identify your blood samples and DNA samples. Your personal information will not be shared with the personnel in the laboratories or anyone outside of Dr. Josiassen’s staff.

If you consent to participate in this study, we will take about 30 minutes of your time. We will obtain 4 tubes of blood from a vein in your arm. The amount of blood is about 3 tablespoons. These tubes will be coded with your unique study ID number.

All blood samples will be sent to the Rutgers University Cell and DNA Repository (the DNA Repository) directed by Dr. Jay Tischfield of the Department of Genetics at Rutgers University. The DNA Repository is sponsored by the National Institutes of Mental Health (NIMH). Your samples will be used to create a “cell line”, which allows the researchers to have a permanently available source of your DNA. Your DNA can then be used for scientific research now and in the future. The DNA Repository will also store the following information: your age, sex, ancestry, medical diagnoses, and medication history. The DNA Repository will not be have any of your personal identifiers, like your name, address or telephone number. In other words, researchers will be able to study your DNA without knowing your identity.

DNA samples will be sent to Patrick Sullivan, MD, at the Center for Psychiatric Genomics at the University of North Carolina, Chapel Hill, North Carolina. Dr. Sullivan and his team will then study your DNA for differences that might help us understand your illness. We estimate that around 5-10% of the individuals we study may be found to have such changes.

Dr. Sullivan will work closely with Dr James Evans, MD, PhD who directs the Clinical Adult and Cancer Genetics Services at the University of North Carolina. Dr. Evans leads NCGENES (North Carolina Clinical Genomic Evaluation by NextGen Exome Sequencing), a genomic study similar to this one but which focuses on other disorders. His team of medical genetic and neuroscience experts will also examine your DNA looking for genetic differences (called “variants”) that could help explain why standard psychiatric treatments have not been very helpful for you. You may learn one of three types of results:

1. ***Positive result:*** a variant has been identified in a gene associated with a genetic disorder that explains why your psychotic symptoms have not responded to treatment.

2. ***Uncertain result*:** a variant has been identified in a gene associated with a genetic disorder that may explain why your psychotic symptoms have not responded to, but the clinical meaning of that variant is not known for certain.

3. ***Negative result:*** no potentially causative variant has been identified in the subset of genes that was studied that explains why your psychotic symptoms have not responded to modern-day treatment.

It is possible that we could find genetic variants that are not related to your psychiatric disorder. This is called ***incidental information***. In very rare cases, incidental information might be found that suggests that you currently have a serious medical condition ***that can be treated*** OR that you are at high risk for a future medical problem ***that can likely be prevented***. This is called ***medically actionable information***. It is unlikely that our team of experts will find this type of information. However, if ***actionable information*** is identified, we will need to have a formal discussion about whether or not you want this new information to be shared with you and your treating doctor. Also, if ***actionable information*** is identified we will want to have another sample of your DNA tested in another separate laboratory to validate the finding.

If you have a medical condition that could make it unsafe for you to have blood drawn (for example, hemophilia), it is important that you tell the research staff so that they may consult with your medical doctor about the safety of your providing a blood sample. You may participate if you know that you have the AIDS virus, ARC (AIDS - related complex), or Hepatitis. This information, like all information obtained in the course of the research project, will be kept strictly confidential.

***10. What are my responsibilities if I take part in this research?***

If you take part in this research, it is very important that you:

- Follow your physician’s or researcher’s instructions.
- Tell your study physician or researcher right away if you have a complication or injury.
- Be as honest and accurate as you can about your medical and psychiatric history.

***11. What happens if I do not want to be in this research?***

You may decide not to take part in the research and it will not be held against you.

***12. What happens if I say yes, but I change my mind later?***

You agree to take part in the research now and stop at any time it will not be held against you. If you decide to leave the research, please notify Dr. Josiassen or the research staff. Your participation may be terminated by Dr. Josiassen or his staff without your consent if you are not able to participate in the procedures or if you are not eligible for the study. You also have the right to request that all your cell lines, any remaining DNA samples, demographic and any results pertaining to your personal DNA be removed from the DNA Registry at Rutgers University. This can be done by contacting the Principal Investigator, Dr. Richard C. Josiassen (267) 304-1115.

***13. Is there any way being in this study could be bad for me?***

The possible risks will usually be small. These include both medical and psychological concerns. There is a small risk in obtaining a blood sample. The amount of blood removed is small (40 ml) and generally will not cause significant circulation problems. You may have mild discomfort and rarely a small bruise, clot, or infection may occur at the site of the blood draw. Some people may become faint or light-headed during or immediately after the blood draw. The blood will be obtained by a trained and experienced phlebotomist who has worked with chronic psychiatric patients for many years, and is certified to obtain blood. The medical risk is low.

There may be psychological risks from participating in a study that is trying to find genetic causes of treatment resistant psychotic symptoms. You could become distressed because you are being studied as a treatment-resistant individual. While we feel that this risk is small, counseling will be offered to you if needed.

There are other important risks to consider. You might be concerned about risk to privacy and confidentiality resulting in discrimination because of your genetic information. A US Federal law called the Genetic Information Nondiscrimination Act (GINA) generally makes it illegal for health insurance companies, group health plans, and most employers to discriminate against you based on your genetic information. GINA does not protect you against genetic discrimination by

companies that sell life insurance, disability insurance, or long-term care insurance. GINA also does not protect you against discrimination based on an already-diagnosed genetic condition or disease.

***14. Do I have to pay for anything while I am on this study?***

There is no cost to you for participating in this study. You will not be charged for any tests specifically required for this research study, but you or your insurance company will still be billed for tests or procedures that are considered “standard of care” and would have been part of your medical treatment even if you did not participate in this study. These treatment costs include but are not limited to drugs, routine laboratory tests, x-rays, scans, surgeries, routine medical care, and physician charges. Your health insurance company may not pay for these “standard of care” charges because you are in a research study. If your insurance company does not pay for costs associated with this research study that are considered “standard of care” for your medical treatment, then you will be billed for these costs. You are responsible for paying for any insurance copays and any deductibles due under your insurance policy, and any charges your insurance company does not pay.

***15. Will being in this study help me in any way?***

We cannot promise any benefits to you or others from your taking part in this research. It is unlikely that our team of experts will find information from your genomic testing that is considered to be actionable. In a minority of cases, new therapeutic approaches might be suggested by the results, or an important medical risk might be uncovered. In case actionable information is identified, you will need to make some important decisions as to whether or not you want to learn this information and if so, whether or not you also wish it to be communicated to the doctor who is treating you. Please think carefully about this decision and indicate your choice by circling one of the following statements.

____________NO. I do not want to be informed about the results of my genomic testing, nor do I want my treating physician to be informed.

____________YES. I want to be informed in writing about the results of my genomic testing. I want the information to be reported back to me ONLY, and then I will decide whether or not to share it with the doctor who treats me. I understand that, in the majority of cases, the DNA findings will not affect my treatment. If actionable information is identified, a medical ethicist who specializes in the ethics of genetics will be present and take part in the discussion of your genomic results.

***16. What happens to the information we collect?***

Efforts will be made to limit your personal information, including research study and medical records, to people who have a need to review this information. We cannot promise complete secrecy. Organizations that may inspect and copy your information include the IRB and other representatives of this organization. Federal law requires that Drexel University College of Medicine and its affiliated researchers, health care providers, and physicians protect the privacy of information that identifies you and relates to your past, present, and future physical and mental health conditions (“protected health information”). If you agree to participate in this study, the study information (which does not contain your identity) may be shared among the study collaborators, an ethical review board for this study, the U.S. Food and Drug Administration and regulatory authorities in other countries. This study information (which does not contain your identity) may also be published. “Protected health information” will be used or shared with others as explained below. Medical records that do contain your identity will be treated as confidential information. They may be inspected by the above mentioned parties, but information will not be taken from Drexel University College of Medicine (for example, by photocopying) except in the case of an audit by the U.S. Food and Drug Administration. In those cases your identifying information would be accessed and disclosed only to the extent absolutely necessary for the audit.

The records are kept locked in file cabinets, identified by study numbers to preserve confidentiality. Your records or any part thereof can only be legally obtained with your written permission specifying what exact information is to be released or if court ordered by law.

***17. Can I be removed from the research without my OK?***

The person in charge of the research study or the sponsor can remove you from the research study without your approval. Your participation may be terminated by Dr. Josiassen or his staff without your consent if you are not able to participate in the procedures or if you are not eligible for the study.

***18. What else do I need to know?***

This research study is being done by Drexel University.

Dr. Josiassen owns Translational Neurosciences LLC. which is sponsoring the phlebotomist to obtain your blood sample.

If you become ill or injured during this study, contact Dr. Richard C. Josiassen at (267) 304-1115. We will get you medical care. If you need care right away, go to the nearest emergency room or call 9-1-1. Inform all medical emergency staff that you are taking part in this study.

If a “research related injury” results from your participation in this research study, medical treatment will be provided. The costs for all your medical treatment will be billed to you and/or your insurance. A “research related-injury” means injury caused by the product or procedures required by the research which you would not have experienced if you had not participated in the research.

Researchers from other institutions will be able to obtain approval from NIMH to study your DNA, demographic and health information stored in the DNA Repository. No personal identifiers will be given to them.. These researchers may work with a private company. Such companies have a financial interest in using information found from studying DNA, for example to develop commercial products that may later help others by improving the diagnosis and treatment of various medical problems. These companies may patent products or sell discoveries based on this research. Some of the scientists who study your DNA, your demographic information, and health information may get some financial benefit from this work. There are no plans to provide any compensation to you or your heirs should this occur.

**Authorization to Use and Disclose Protected Health Information**

Federal law provides additional protections of your personal information that are described here.

***A. Individually Identifiable Health Information That Will Be Collected***

The following personal health information about you will be collected and used during the research study and may be given out to others:

- Your date of birth.
- Personal medical and psychiatric history.
- Information from blood tests.
- Information in medical records located in your doctor’s office or at other medical facilities you may have received treatment.

***B. Who Will See and Use Your Health Information within Drexel University***

The researcher and other authorized individuals involved in the research study at Drexel University will see your health information during and may give out your health information during the research study. These include the researcher and the research staff, the institutional review board and their staff, legal counsel, research office and compliance staff, officers of the organization and other people who need to see the information in order to conduct the research study or make sure it is being done properly. Your health information may be disclosed or transmitted electronically.

***C. Who Else May See and Use your Health Information***

Other persons and organizations outside of Drexel University may see and use your health information during this research study. These include:

- Governmental entities that have the right to see or review your health information, such as The Office for Human Research Protections, and the Food and Drug Administration
- Doctors and staff associated with the research study.
- Staff at the NIMH-sponsored Rutgers University Cell and DNA Repository (RUCDR) under the direction of Dr. Jay Tischfield, Chair of the Department of Genetics at Rutgers University.
- Dr. Patrick Sullivan and his staff at the University of North Carolina at Chapel Hill who will direct the genomics study.
- Dr. James P. Evans and staff at the Clinical Adult and Cancer Genetics Services at the University of North Carolina.
- Members of the study’s Clinical Advisory team.

If your health information is given to someone not required by law to keep it confidential, then that information may no longer be protected, and may be used or given out without your permission.

***D. If you do not want to give authorization to use your health information***

You do not have to give your authorization to use or give out your health information. However, if you do not give authorization, you cannot participate in this research study.

***E. How to cancel your authorization***

At any time you may cancel your authorization to allow your health information to be used or given out by sending a written notice to Human Research Protection at. 1505 Race Street,7th Floor Bellet Building, Philadelphia, Pennsylvania, 19102. If you leave this research study, no new health information about you will be gathered after you leave. However, information gathered before that date may be used or given out if it is needed for the research study or any follow-up.

***F. When your authorization ends***

Your authorization to use and give out your health information will end when the research study is finished.

***G. Your right to inspect your medical and research records***

You have the right to look at your medical records at any time during this research study. However, the researcher does not have to release research information to you if it is not part of your medical record.

***H. Information about Genetic Information Nondiscrimination Act (GINA)***

The Federal law, called the Genetic Information Nondiscrimination Act (GINA), generally makes it illegal for health insurance companies, group health plans, and most employers to discriminate against you based on your genetic information. This law generally will protect you in the following ways:

- Health insurance companies and group health plans may not request your genetic information that we get from this research study.
- Health insurance companies and group health plans may not use your genetic information when making decisions regarding your eligibility or premiums.
- Employers with 15 or more employees may not use your genetic information that we get from this research study when making a decision to hire, promote, or fire you or when setting the terms of your employment.

However, GINA will not protect you if you already have a genetic disease or disorder and does not prohibit discrimination on the basis of an existing genetic disease or disorder. In addition, this new Federal law does not protect you against genetic discrimination by companies that sell life insurance, disability insurance, or long-term care insurance.

**Permission to Take Part in a Human Research Study**

**Signature Block for Capable Adult**

Your signature documents your permission to take part in this research.

**DO NOT SIGN THIS FORM AFTER THIS DATE**

**___________________________________________ __________________**

Signature of subject Date

**___________________________________________**

Printed name of subject

**___________________________________________ __________________**

Signature of person obtaining consent Date

**___________________________________________**

Printed name of person obtaining consent

My signature below documents that the information in the consent document and any other written information was accurately explained to, and apparently understood by, the subject, and that consent was freely given by the subject

**___________________________________________ __________________**

Signature of witness to consent process Date

**___________________________________________ __________________**

Printed name of person witnessing consent process

**Permission to Take Part in a Human Research Study**

**Signature Block for Adult Unable to Consent**

**DO NOT SIGN THIS FORM AFTER THIS DATE**

**___________________________________________**

Printed name of subject

I am willing to serve as a legally authorized representative for the above named subject. The investigators have explained to me the role and responsibilities of a legally authorized representative. My signature documents my permission for the above named subject to take part in this research.

**___________________________________________ __________________**

Signature of legally authorized representative Date

**___________________________________________**

Printed name of person obtaining consent

**______________________________________________________________________________**Address

**______________________________________________________________________________**City, State, ZIP

**_____________________________________ ______________________________**

Phone Email

| Highest | The following individuals in descending order of priority are capable of serving as a legally authorized representative (LAR). Check the category that best describes the LAR’s relationship to the subject. |
| --- | --- |
|  | Health care agent appointed by the subject in a Power of Attorney; |
|  | Court-appointed guardian authorized to consent to the subject’s participation in the protocol in a current court order issued within the subject’s jurisdiction; |
|  | Spouse or domestic partner (unless an action for divorce is pending) and adult children of the subject who are not the children of the spouse or domestic partner; |
|  | Adult child; |
|  | Natural or adoptive parent; |
|  | Adult brother or sister |
|  | Adult grandchild |
| Lowest | Adult who has knowledge of the subject’s preferences and values, including, but not limited to, religious and moral beliefs, to assess how the principal would make health care decisions. Unless related by blood, marriage, or adoption, the adult may not be the principal’s attending physician or other health care provider nor an owner, operator or employee of a health care provider in which the principal receives care. |

**___________________________________________ __________________**

Signature of person obtaining consent Date

**___________________________________________**

Printed name of person obtaining consent

My signature below documents that the information in the consent document and any other written information was accurately explained to, and apparently understood by, the subject, and that consent was freely given by the subject.

**___________________________________________ __________________**

Signature of witness to consent process Date

**___________________________________________**

Printed name of person witnessing consent process

Assent

 Obtained

 Not obtained because the capability of the subject is so limited that the subject cannot reasonably be consulted.
